# Supplementary material for: Sequencing Reveals miRNAs Enriched in the Developing Mouse Enteric Nervous System
Source: Noncoding RNA. 2023 Dec 22;10(1):1. doi: 10.3390/ncrna10010001 (PMC10801555; doi:10.3390/ncrna10010001)
Supplement: Supplementary file 1 [file ncrna-10-00001-s001.zip › revisedSupplement/tableS1.docx]

**Table S1 Extended animal, experiment, and housing information**

| **Animal Information** |  |
| --- | --- |
| *Species* | *Mus musculus* |
| *Age* | P28 weaning, P56-84 for bowel harvesting |
| *Sex* | Male & female (identified when possible) |
| *Strain* | See Methods for full list |
| *Genetic background* | 129S6/SvEvTac or C57BL6/J; see Methods |
| *Type of genetic modification* | Flanking by loxP sites around *Mir137* locus paired with multiple *cre*-expressing transgenes |
| *Genotypes used* | Varies; see Methods |
| *Microbe status* | Specific pathogen free |
| *Origin* | In-house breeding |
| *Weight* | Varies; see Results |
| *Procedure history* | None |
| *Drug & test naivete* | No exposure prior to experiments |
| *Health & immune status* | Healthy |
| *Quarantine & acclimatization* | N/A |
| **Housing** |  |
| *Type of facility* | Specific pathogen free |
| *Light/dark cycle* | 12h/12h, 18:00-06:00 dark |
| *Type of lighting* | Fluorescent ceiling bulbs |
| *Facility temperature* | 72°F +/- 2°F |
| *Facility humidity* | 30-70% |
| *Ventilation* | Room air changes 10-15x/hr, individually ventilated cage racks of ~70 air changes per hour |
| *Noise* | Ongoing construction in other parts of animal facility & adjacent building lot |
| *Type of cage* | Tecniplast GM500 |
| *Size of cage* | 77.66in^2^ |
| *Bedding material* | ¼” corncob or ¼” corncob/ALPHA-dri® blend |
| *Number of cage companions* | 1-5; singly housed if sole male in litter or after mating |
| *Presence & type of enrichment* | Present; nestlet material with mouse hut and/or shelf |
| *Frequency of cage change* | Weekly initially, every other week as of January 2022 |
| *Frequency of handling* | Solely for cage changes and experiments |
| *Breeding program* | Couple or trio matings; sibs or cousins mated together |
| *Type of food* | Lab Diet 5015 |
| *Food composition* | >11% fat, <3% fiber |
| *Food pre-treatment* | None |
| *Food & water availability* | Ad libitum |
| *Type of water* | Reverse osmosis purified, pH 7 |
| *Water pre-treatment* | None |
| *Frequency of water change* | Automated system |
| *Water delivery (bottle vs automatic)* | Automatic |
